# Practical Notes on Diagnosis and Treatment

**Published:** 1907-05-11

**Authors:** 


### PRACTICAL NOTES ON DIAGNOSIS AND TREATMENT.

**Hiccough.**

A BAD and obstinate hiccough can sometimes be checked by an occasional full pinch of tobacco snuff.

**Lemonade for Diabetes.**

THE following is a useful beverage to check thirst in patients suffering from diabetes: Pure glycerine, ʒvj.; citric acid ʒiss.; water, 2 pints.

**Skin Eruptions from Drugs.**

NEVER forget that anomalous rashes, papular, bullar, or pustular, sometimes accompanied with ulceration and granuloma, which look like lupus or malignant disease, may all be caused by iodide of potassium in medicinal doses.—*Dr. Pye-Smith.*

**Heart Disease in Stout Patients.**

IN all cases of chronic heart disease where the patient is stout, the first thing to do is to get rid of the fat. When this is accomplished the heart has less work to do, and both the circulation and respiration are relieved.—*Dr. Harry Campbell.*

**Cough in Chronic Bronchitis.**

THE distressing cough from which many patients with chronic bronchitis suffer on waking after a night's rest, can often be checked, and the expectoration facilitated, if the patient is instructed to sip a mixture made by adding together of equal parts of boiling milk and potash water.

**Laryngeal Papilloma.**

THIS occurs either in a solitary or a multiple form. It is met with at all periods of life, and, particularly in children, it is almost the only form of benign laryngeal growth (except a few rare cases of fibromata and cysts) that comes under observation.—*Sir Felix Semon.*

**Piperazin.**

FOR the relief of obscure pains in gouty people piperazin is almost indispensable. Especially is it of value in a form of enteralgia, probably dependent on duodenal dyspepsia and common in gouty subjects. This is a most painful and intractable affection, but to piperazin (grs. v.), combined with alkalies and carminatives, it seldom fails to yield.—*Dr. Jas. Cagney.*

**Tabes Dorsalis.**

REST in bed I believe to be efficacious in cases of this disease. I do not think the patient ought to be kept solely at rest in bed; there ought to be combined with this, very active treatment of the spine by counter-irritation by means of the actual cautery. The best results I have seen in tabes in hospital have been in cases in which the cautery has been used very freely indeed, this treatment being accompanied by rest in bed.—*Dr. James Taylor.*

**Extra-uterine Pregnancy.**

DIRECTLY you make a diagnosis of extra-uterine gestation you must operate, whatever the condition of affairs is. I think it may be said that there is no exception to that rule.—*Dr. W. R. Dakin.*

**Hyperhidrosis.**

IN a case of hyperhidrosis consecutive to influenza, in which many remedies were tried without success, the hypodermic injection of picrotoxin, $\frac{1}{46}$ grain daily, continued for a week, completely checked the sweating.

**Thymol in Toothache.**

IN toothache arising from a hollow tooth thymol has been advised. The cavity is filled with a tuft of cotton-wool on which a few grains of thymol have been sprinkled. If any irritation of the mucous membrane is caused it can be checked by rinsing the mouth with warm water.

**Belladonna in Renal Colic.**

DR. W. MURRAY administers tincture of belladonna in attacks of renal colic. He gives it in doses of 30 to 40 minims every two to three hours until slight delirium sets in. This production of the physiological evidences of the action of the drug is essential to success; it is usually followed by cessation of the pain and passage of the calculus.

**Præcordial Pain.**

WHILE præcordial pains in young people are usually of trifling importance and do not signify heart disorder, in senile persons the persistence of a sort of præcordial pain has often a very serious meaning; they may die as suddenly with pain which they merely call dyspeptic—pains which you also call dyspeptic—as if they had what you call "typical" angina pectoris.—*Professor Clifford Allbutt.*

**Bi-lateral Sciatica.**

TRUE "sciatica" is rarely bi-lateral. When complained of it is sometimes found to be an evidence of tabes dorsalis or of some other disease of the spinal apparatus; in other instances it is associated with diabetes mellitus, and its presence ought always to call for a careful examination of the urine. Another possible explanation is a tumour or other cause of pressure within the pelvis.

**Furunculosis.**

THE best way to treat furunculi is to open every boil as soon as it appears, syringe it out with some carbolic lotion—1 in 60—or rub in iodoform. Never poultice a boil with linseed-meal or bread, but if there is great pain you may apply an antiseptic one, such as a warm boracic fomentation. But the best plan is to open each boil and clear it out as soon as possible.—*Dr. Radcliffe Crocker.*

